# Patents on Endophytic Fungi Related to Secondary Metabolites and Biotransformation Applications

**DOI:** 10.3390/jof6020058

**Published:** 2020-05-01

**Authors:** Daniel Torres-Mendoza, Humberto E. Ortega, Luis Cubilla-Rios

**Affiliations:** 1 Laboratory of Tropical Bioorganic Chemistry, Faculty of Natural, Exact Sciences and Technology, University of Panama, Panama 0824, Panama; dtorresm.507@gmail.com (D.T.-M.); humberto.enrique.ortega@gmail.com (H.E.O.); 2 Vicerrectoría de Investigación y Postgrado, University of Panama, Panama 0824, Panama; 3 Department of Organic Chemistry, Faculty of Natural, Exact Sciences and Technology, University of Panama, Panama 0824, Panama

**Keywords:** endophytic fungi, patents, secondary metabolites, biotransformation, biological activity

## Abstract

Endophytic fungi are an important group of microorganisms and one of the least studied. They enhance their host’s resistance against abiotic stress, disease, insects, pathogens and mammalian herbivores by producing secondary metabolites with a wide spectrum of biological activity. Therefore, they could be an alternative source of secondary metabolites for applications in medicine, pharmacy and agriculture. In this review, we analyzed patents related to the production of secondary metabolites and biotransformation processes through endophytic fungi and their fields of application. We examined 245 patents (224 related to secondary metabolite production and 21 for biotransformation). The most patented fungi in the development of these applications belong to the *Aspergillus*, *Fusarium*, *Trichoderma*, *Penicillium*, and *Phomopsis* genera and cover uses in the biomedicine, agriculture, food, and biotechnology industries.

## 1. Introduction

The term endophyte refers to any organism (bacteria or fungi) that lives in the internal tissues of a host. This endophyte–host association is complex: it is normally driven without causing harm or apparent disease symptoms and provides benefits in survival, fitness, biodiversity, and ecosystem function for both parties by enhancing the response to environmental stress and producing the same or similar compounds that originate in the host [1,2,3]. In particular, fungal endophytes have been the focus of many studies due to their prospective promise in the production of secondary metabolites with pharmacological, agricultural, industrial, or biotechnological applications [4,5,6].

Endophytic fungi were discovered over a century ago; however, it was not until about three decades ago, with the discovery of the taxol-producing endophytic fungus *Taxomyces andreanae*, that they gained remarkable relevance due to the abovementioned production of active secondary metabolites [7,8,9,10].

As was the case with taxol, the process for the isolation and purification of metabolites in adequate yields remains a major concern; low yields due to the exploitation of the host for the extraction process of metabolites are also associated with environmental impacts, and new strategies such as involving the use of endophytic microorganisms instead of the host themselves have offered new niches that should be meticulously investigated and used as a base for sustainable research and development [11,12].

The present review covers patents related to the production of natural products with biomedical and agricultural applications using endophytic fungi, enabling the development of new lead compounds in the process of finding new drug candidates or precursors for the synthesis of new molecules. We also cover the production of secondary metabolites in biotransformation processes by using endophytic fungi.

## 2. Materials and Methods

This review was conducted mainly through searches of the databases Scifinder^®^ and Google Patents. Our search was made under the subjects “endophytic fungi” and “patents” covering the period from 2001 to 2019. 4670 references were found. After removing duplicates, we selected those related to the production of secondary metabolites and biotransformation. Resulting in 245 documents from which 224 were related to any kind of secondary metabolite derived from endophytic fungi and 21 detail biotransformation processes of metabolites through endophytic fungi. The patents covered in this study are described in Table 1 and Table 2. 

## 3. Results

The description and analysis of patents was divided into two sections: those that are connected to the production of secondary metabolites and those associated with biotransformation processes. Likewise, two tables were constructed in which the main generalities of each patent are summarized.

### 3.1. Production of Secondary Metabolites

**Figure 1:**
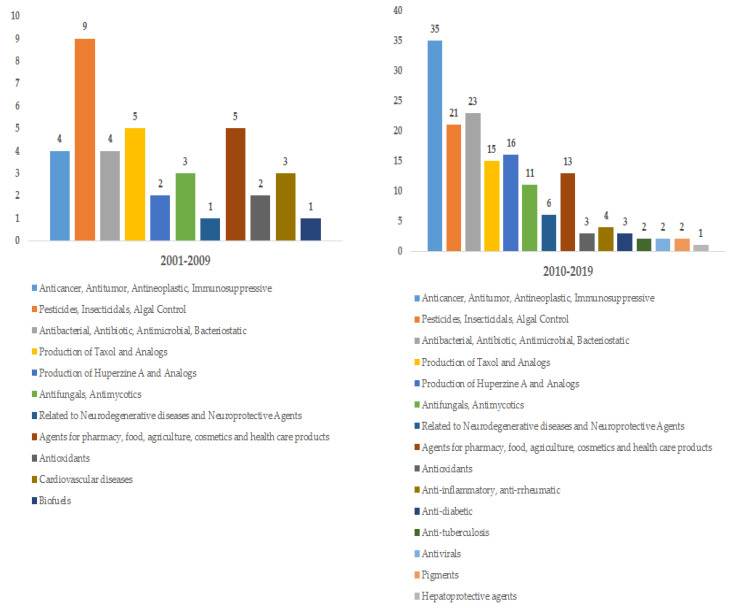
Progression on the patents and fields of application in the periods 2001–2009 compared to 2010–2019. x-axis year; y-axis numbers of patents.

Early patents consisted mainly of registering the endophytic strains capable of producing specific compounds or those that represented a novel source of active metabolites (chanoclavine in EP1142986A2; resveratrol in CN1948459A; gallic acid in CN101280279A; taxol in CN101486974A) and very few applications. However, over time, patents were developed to include the registration of methods and procedures to produce and recover the compounds of interest (with a known biomedical application) or to optimize or increase their production (podophyllotoxin in US20040248265A1; taxol in CN1624103A; camptothecin in US20060134762A1; huperzine A in CN101275116A). In the last ten years, patents have been focused on using novel or enhanced fermentation processes to obtain high yields of products and provide possible applications for the metabolites (alpha-pyrone in CN110563740A; epimedins A–C in CN110511876A; differanisole A in CN109971655A; 5, 8-ergosterol epoxide in CN109971651A). The distribution of the patents in relation with the principal areas of application are illustrated in Figure 1. The production of taxol and huperzine A were considered as other application outside of their anticancer and anti-Alzheimer property respectively, due to the number of patents and economic importance.

The principal applications consist on providing metabolites that are precursors of bioactive molecules (baccatin III and cephalomannine in CN103194502A) and those that can be use as anticancer, antitumor, antineoplastic or immunosuppressive agents (anthraquinone compounds in CN102586355A; cerrenin D in CN109456191A; alterporriol P in CN102633616A; dalesconol A and B in CN104031948A; quinazoline alkaloid compound in CN103570744A); in pesticides, insecticidal, algal control (diterpene alkaloid-like compounds in CN102190699A); as antibacterial, antibiotic, antimicrobial, bacteriostatic (beauvericin in CN101240249A; diterpene alkaloid compound in CN102190612A); as antifungal and antimycotics (*Trichoderma* acid in CN103083290A); in neurodegenerative diseases and neuroprotective agent (huperzine A in CN102191294A); as agents in pharmacy, food, cosmetics, agriculture and health care products (pseutorin A in CN104774774A; alterlactone in CN110093383A); antioxidant (flavipin in CN103087923A); anti-inflammatory and anti-rrheumatic (1,4-napthoquinones in CN109293494A); in cardiovascular diseases (breviscapine in CN1421522A); anti-diabetes (2 isabolene sesquiterpenes in CN109096056A); anti-tuberculosis (enniatin compounds in CN101669939A); antiviral (alterporriol Q and R in CN102643186A); as pigments; hepatoprotective agents (pyrrole-type compounds in CN103667073A); in biofuels. Table 1 displays the patents, endophytic fungi, host organism, secondary metabolites, and disclosed applications. The structures of the compounds listed in Table 1 and Table 2 are shown in Figure S1 (see the Supplementary Information).

The principal endophytic fungi reported in this section of patents belong to the genera *Aspergillus*, *Fusarium*, *Trichoderma*, *Penicillium,* and *Phomopsi*s with 31, 24, 18, 16, and 8 patents, respectively, and compounds such as taxol or paclitaxel, huperzine A, camptothecin, podophyllotoxin, and resveratrol. Methods for enhancing their production represented most of the registered applications. Furthermore, the diversity of compound structures demonstrates the capability of fungi to synthetize simple or very complex molecules.

Mostly, *Aspergillus* endophytes from plants of the genera *Taxus* and *Torreya* are described as having applications related to obtaining the highest yield of paclitaxel or its precursors, like baccatin III and cephalomannine, due to their anticancer activity. Endophytes from *Huperzia serrata* have been linked to the production of huperzine A and its analogs due to their anti-senile dementia and anti-neurodegenerative applications. Plant endophytes such as *Nothapodytes nimmoniana* and *Camptotheca acuminata* have been linked to the production of the antineoplastic agent camptothecin and some analogs. Production of the lignan-type compound podophyllotoxin has been described for several endophytes. This compound has high biomedical potential as an anticancer, antiviral, and antibacterial agent, among others, and is the precursor of the anticancer drugs etoposide and teniposide. The stilbenoid compound found on grape skin, resveratrol, could have promising therapeutic actions against obesity, type II diabetes mellitus, metabolic syndrome, cancer, autism, dementia, and Alzheimer’s disease [13]. Therefore, a number of patents involving endophytes of the genera *Cladosporium*, *Fusarium*, *Alternaria,* and *Penicillium* for its production were registered. The demand for natural resveratrol has gained traction in various end-use industries.

### 3.2. Biotransformation by Endophytic Fungi

Biotechnological processes enable the production of useful molecules with a decrease in the generation of pollutants, reducing the use of solvents and reagents, minimizing the consumption of energy, and providing a way to obtain active compounds with greater specificity and efficiency. The use of endophytic fungi in biotechnological processes, such as biotransformation, is in its early stages of development and has some limitations [238]. However, there have been some reports of fungi that have been used in biotransformation [239,240,241,242].

Table 2 lists a group of patents that illustrate the efforts toward using endophytic fungi to obtain molecules of biological importance such as the ginsenosides [243] and glycyrrhetinic acid monoglucuronide [244].

Fungi from the genera *Absidia*, *Zygorhynchus*, *Xylaria,* and *Fusarium* have been patented to obtain ginsenoside Rd by the transformation of ginsenoside Rb1. Fungi from the genera *Microsphaeropsis*, *Aspergillus*, and *Chaetomium* have been patented for the biotransformation of glycyrrhizinic acid into glycyrrhetinic acid monoglucuronide.

## 4. Discussion

The study of endophytic fungi as a source of bioactive secondary metabolites has its first beginnings in 1993 with the discovery of taxol [4], until then, the primary sources of active natural molecules were isolated mainly from plants [266]. About two decades ago, the study of endophytic fungi as producers of active molecules has been emphasized due to obtaining compounds originally produced by plants or due to the production of novel secondary metabolites [11,267], Thus, fungi from genus *Aspergillus*, *Fusarium*, *Penicillium* and *Pestalotiopsis* has been recognized as producers of anticancer compounds and having pharmaceutical potential [12,268]. It is estimated that only around 1% of the microorganisms have been cultivated, and within this groups, endophytic fungi corresponded to the least studied [269].

**Figure 2:**
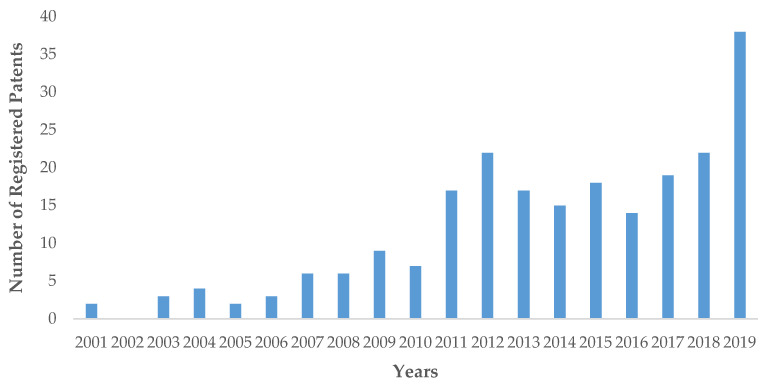
Number of registered patents from 2001 to 2019 linked to endophytic secondary metabolites and biotransformation processes through endophytic fungi.

Through this review, we have demonstrated the wide number of endophytic fungi involved in the development of methods and techniques for the application of isolation and fermentation to obtain secondary metabolites with high potential and applications in biomedicine, agriculture, and biotechnology processes. Figure 2 shows the number of patents registered for secondary metabolites and biotransformation processes through endophytic fungi for the period from 2001 to 2019. We found 224 patents related to secondary metabolites and 21 patents related to biotransformation. *Aspergillus*, *Fusarium*, *Trichoderma*, *Penicillium,* and *Phomopsis* were the most representative genera for secondary metabolites. 

**Figure 3:**
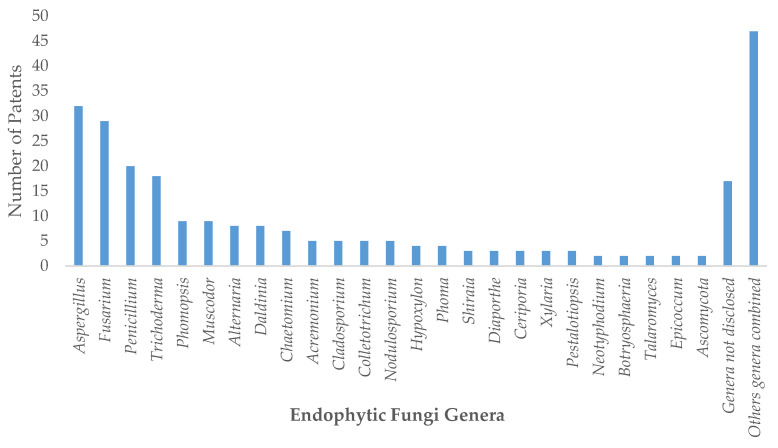
Number of patents reported for various endophytic fungi by genera.

*Fusarium* and *Penicillium* were the most commonly registered endophytic fungi genera among the 21 patents reviewed for biotransformation processes. Figure 3 shows the number of patented genera. The most notable applications patented were antimicrobial, antibacterial, anticancer, and those related to neurodegenerative diseases. For biotransformation processes, the conversion of ginsenosides and glycyrrhizinic acid were the most patentable applications due to their importance and potential in the pharmaceutical and food industries.

Table 1 and Table 2 showed that the majority of the endophytic fungi were derived from plants, but we could also find patents where the host was soft corals or insects.

The global market for compounds like taxol is expected to reach USD $99 million by 2021 [270], and for resveratrol, the projected growth from 2018 to 2028 in revenue terms is 8.1% from USD $97.7 million [271]. Under the objectives of the 1992 Convention on Biological Diversity for the sustainable use of its components and the Nagoya Protocol on Access to Genetic Resources and the Fair and Equative sharing of benefits derived from the use of genetic resources [272], endophytic fungi and their derived compounds could open a new set of industries and economics in development countries with high biodiversity for the low-cost yield of high-profit molecules that can be applied in the fields discussed in this review. 

## Figures and Tables

**Table 1:** Endophytic fungi and their methods of production of natural products.

Patent No.	Endophyte	Host ^1^	Patent Details	Ref.
EP1142986A2	*Neotyphodium* sp.	Not disclosed	Chanoclavine (1)-production.	[14]
US6329193B1	*Cladosporium macrocarpon*	*Taxus* spp.	Production of taxol.	[15]
CN1421522A	*Alternaria* sp.	*Erigeron* sp.	Production of breviscapine B (2) and other flavonoids for the treatment of cardiovascular diseases and for preparing antitumor medicine.	[16]
US6638742B1	*Alternaria* sp.	*Alnus rubra*, *Corylus* sp., *Cytisus scoparius*, *Ginkgo* sp.	Methods for obtaining and recovering taxanes, including paclitaxel (3), from novel sources.	[17]
US6613738B1	*Cryptosporiopsis* cf. *quercina*	*Tripterigeum wilfordii*	Isolation of cryptocandin possessing antifungal activity.	[18]
US20040185031A1	*Muscodor vitigenus*	*Paullinia paullinioides*	Novel fungi that produces naphthalene and applications.	[19]
US20040206697A1	*Muscodor albus*	Cinnamon tree	Novel fungi and production of organic volatile antibiotics effective in the treatment of human and animal waste.	[20]
US20040248265A1	*Phialocephala fortinii*	*Podophyllum* sp.	Identification of podophyllotoxin-producing fungi and methods for recovering podophyllotoxin (4) from such fungi.	[21]
WO2004106487A2	*Neotyphodium lolii*	Pooideae grass	Production of janthitrem epoxide (5) compounds in combination with ryegrass instead of compounds that affect the health and performance of grazing animals.	[22]
CN1624103A	Mix of *Taxus* endophytes	*Taxus chinensis*	Increase the production of taxol and taxol precursors.	[23]
US6911338B2	*Muscodor* sp.	*Cinnamomum zeylanicum*, *Grevillea pteridifolia*	Production of organic volatile antibiotics with activity on specific plant pathogens, bacteria, nematodes and insects.	[24]
CN1850765A	*Halorosellinia* sp.	mangrove	Obtaining quinone compounds (6–7) with antitumor activity.	[25]
US20060134762A1	Fungal strain MTCC 5124	*Mappia* sp.	New source in the form of a novel endophytic fungal strain for the production of camptothecin (8) and camptothecinioids and an improved process for producing these.	[26]
US7070985B2	*Muscodor albus*	*Cinnamomum zeylanicum*	Novel fungi and production of organic volatile antibiotics effective in the treatment of human and animal waste products.	[27]
CN1896232A	*Fusarium* sp.	*Ginkgo biloba*	Production of plasmin.	[28]
CN1948459A	*Cladosporium* sp.	*Parthenocissus tricuspidatae*	Production of resveratrol (9).	[29]
CN1951907A	*Aspergillus niger*	*Euphorbia* sp.	Preparation of compound 2,3-diamino-6-hydroxy-benzoic acid-2-ethyl-hexyl ester (10), including method, and its application in pharmacy.	[30]
CN101037656A	*Trichoderma harzianum*	*Ilex cornuta*	Preparation of the sesquiterpenoids trichotec-9-en-4-ol, 12, 13, epoxy-, and 4β-acetate (11) as pesticides.	[31]
CN101041840A	*Trichoderma harzianum*	*Ilex cornuta*	Preparation of the sesquiterpenoids trichotec-9-en-4-ol, 12, 13, epoxy-, and 4β-acetate as pesticides.	[32]
US7192939B2	*Pestalotiopsis microspora*	*Terminalia morobensis*	Novel fungi strains capable of producing novel antioxidant and antimycotic agents	[33]
CN101195804A	*Acremonium endophytium*	*Huperzia serrata*	Production of huperzine A (12) analogs through strain liquid fermentation of the endophytic fungi.	[34]
CN101234951A	*Aspergillus clavatonanicus*	mangrove	Production of biphenyl compound (13) including preparation method and application.	[35]
CN101275116A	Mix of endophytes	*Huperzia serrata*	Preparation of huperzine A.	[36]
CN101240249A	*Fusarium* sp.	*Dioscorea zingiberensis*	Production of beauvericin (14) description of its antibacterial activity.	[37]
CN101280279A	*Phomopsis* sp.	*Acer ginnala*	Production of gallic acid (15).	[38]
US7341862B2	*Muscodor albus*	*Cinnamomum zeylanicum*	Novel fungi and production of organic volatile antibiotics effective in the treatment of human and animal waste products.	[39]
CN101412971A	*Fusarium* sp.	*Paris polyphylla* var. *yunnanensis*	Production of 5α, 8α-ergosterol peroxide-6, 22-diene-3β-ol (16), ergosterol-8(9), 22-diene-3β, 5α, 6β, 7α-tetraol (17), and succinic acid (18) as antimicrobial active ingredients.	[40]
CN101468977A	*Phomopsis* sp.	*Azadirachta indica*	Novel pseudo-phomallactone (19) antibacterial compound from fermentation products of an endophytic fungus strain.	[41]
CN101468996A	*Phomopsis* sp.	*Azadirachta indica*	Source of ten-membered lactone 7α-acetoxy-multiplolide A (20) and its applications.	[42]
CN101481379A	*Chaetomium globosum*	*Ginkgo biloba*	Obtaining chaetomugilin D (21) from an acetic acid ethyl ester extract of fermentation liquor.	[43]
CN101486974A	*Aspergillus niger*	*Taxus cuspidata*	Production of taxol from endophytic fungus.	[44]
CN101503658A	Not disclosed	Locoweed	Separation of an endophytic fungus producing swainsonine (22).	[45]
CN101525611A	*Fusarium* sp.	*Chrysanthemum* sp.	Plasmin preparation.	[46]
CN101586082A	*Aspergillus candidus*	*Taxus x media*	Production of taxol. A method for preparing taxol is also given.	[47]
US20090142816A1	*Gliocadium* sp.	*Eucryphia cordifolia*	Production of volatile compounds and hydrocarbons to generate biofuels.	[48]
CN101619291A	*Chaetomium cupreum*	*Macleaya cordata*	Preparation of 3,3′ 6,6′-tetrahydroxy-4,4′-dimethyl-1,1-bi(cyclohexa-3,6-diene)-2,2′,5,5′-tetraone (23) with antitumor properties.	[49]
CN101669939A	Not disclosed	Mangrove	Enniatin compound (24) that aids in the preparation of anti-tubercle drugs.	[50]
CN101701230A	*Fusarium proliferatum*	Mangrove	Improving the output of anticancer anthraquinone compound (25) by utilizing different vaccination methods.	[51]
CN101875905A	*Shiraia bambusicola*	*Phyllostachys edulis* seed	High-yield hypocrellin-producing strain that carries out hypocrellin (26) production by fermentation.	[52]
CN101914452A	*Penicillium chrysogenum*	Not disclosed	Huperzine A-producing strain.	[53]
KR2010104252A	*Scolecobasidium tshawytschae*	Soybean	Gibberellin (27) production using soybean endophyte.	[54]
WO2010062159A1	*Aspergillus* sp.	*Garcinia scortechinii*	Cyclic peptides with utility in anticancer treatments.	[55]
CN101942393A	*Shiraia* sp.	*Huperzia serrata*	Production of huperzine A.	[56]
CN102080110A	Not disclosed	*Nothapodytes nimmoniana*	Technical process for synthesizing a camptothecin sugar derivative.	[57]
CN102080111A	Not disclosed	Icacinaceae plant	Method for endophyte induction to produce 10-hydroxy camptothecin (28).	[58]
CN102080112A	Not disclosed	Icacinaceae plant	Method for endophyte induction to manufacture of 9-methoxycamptothecin (29).	[59]
CN102154116A	*Phomopsis wenchengensis*	Not disclosed	Manufacture of agricultural fungicide (30).	[60]
CN102168017A	*Colletotrichum gloeosporioides*	*Huperzia serrata*	High-producing strain and method for huperzine A production.	[61]
CN102187870A	*Aspergillus oryzae*	Red algae *Heterosiphonia* sp.	Use of diterpene alkaloid (31) secondary metabolites as pesticides.	[62]
CN102190612A	*Aspergillus oryzae*	Red algae	Preparation of diterpene alkaloid (32) with bacteriostatic activity that can be used for preparing antimicrobial agents.	[63]
CN102190614A	*Aspergillus oryzae*	Red algae *Heterosiphonia* sp.	Use of diterpenoid alkaloid (33) as an insecticide agent.	[64]
CN102190698A	*Aspergillus oryzae*	Marine algae	Preparation and application of alga endophytic fungi diterpenoid alkaloid compound (34).	[65]
CN102191294A	*Acremonium endophytium*	*Huperzia serrata*	Production of huperzine A as an anti-senile dementia pharmaceutical ingredient.	[66]
CN102190699A	*Aspergillus oryzae*	Marine algae	Preparation of a diterpene alkaloid-like compound (35) for use as an insecticide.	[67]
CN102220247A	*Verticillium dahlia*	*Radix glycyrrhizae*	Production of glycyrrhetic acid (36).	[68]
IN2010DE00131A	*Aspergillus elegans*	*Asparagus racemosus*	Production of antimicrobial and anticancer lactone metabolite, including an outline of the process.	[69]
JP2011051953A	*Diaporthe* sp.	*Curcuma* sp.	Manufacture of neohexa-hydro-curcumin (37).	[70]
WO2011146634A1	*Hypoxylon* sp.*/Nodulisporium* sp.*/Daldinia* sp.*/Muscodor* sp.	*Persea indica*	Production of volatile organic compounds from these fungi.	[71]
CA2766412A1	*Fungal endophytes of Pinus strobus*	*Pinus strobus*	Antifungal metabolites (38–44).	[72]
CN102321545A	*Penicillium steckii*	*Trypterigium wilfordii*	Production of triptolide (45).	[73]
CN102417883A	*Phomopsis* sp.	*Camptotheca acuminata*	Production and method for preparation of camptothecin.	[74]
CN102464634A	*Trichoderma atroviride*	*Cephalotaxus fortunei*	New compound (46) in secondary metabolites of *C. fortunei* endophytic fungi and its preparation method and application thereof.	[75]
CN102559517A	*Fusarium* sp.	*Podophyllum hexadrum*	Preparation of podophyllotoxin.	[76]
CN102586355A	*Fusarium proliferatum*	Mangrove	Method for producing anticancer anthraquinone compounds.	[77]
CN102628018A	*Aspergillus niger*	*Schisandra chinensis*	Improved production of the main components schisandrol A (47), schisantherin A (48), deoxyschizandrin (49), schisandrin B (50) from *S. chinensis* through fermentation.	[78]
CN102633616A	*Alternaria* sp.	*Sarcophyton* sp.	Preparation of the anthraquinone dimer alterporriol P (51) as an antineoplastic agent.	[79]
CN102643167A	*Aspergillus versicolor*	Marine algae	Fermentation preparation and application as an antibacterial and insecticidal agent of albican-11,14-diol (52).	[80]
CN102643186A	*Alternaria* sp.	*Sarcophyton* sp.	Preparation of the anthraquinone dimers alterporriol Q (53) and alterporriol R (54) for antiviral drugs.	[81]
CN102643755A	*Penicillium chrysogenum*	*Glycyrrhiza glabra*	Endophytic fungus that improves the content of glycyrrhetinic acid by fermenting licorice.	[82]
CN102653720A	*Colletotrichum gloeosporioides*	*Huperzia serrata*	Endophytic fungus capable of generating huperzine A.	[83]
CN102660466A	*Aspergillus penicillioides*	*Schisandra chinensis*	Improves the content of the active ingredients of *S. chinensis:* schizandrin, schisantherin, deoxyschizandrin, and schisandrin B.	[84]
CN102660467A	*Fusarium oxysporum*	*Glycyrrhiza glabra*	Fungal strain that produces glycyrrhetinic acid.	[85]
CN102676392A	*Trichoderma atroviride*	*Salvia miltiorrhiza*	Endophytic fungus that aids in the production of tanshinone I (55) and tanshinone IIA (56).	[86]
CN102701935A	*Trichoderma longibrachiatum*	Seaweed	Preparation of tetranuclear diterpenoid (57) with pesticidal and bacteriostatic activity.	[87]
CN102703327A	*Cladosporium* sp.	*Aconitum leucostomum*	Fungal strain capable of synthesizing aconitine (58) for the preparation of antitumor, anti-inflammatory, and antirheumatic drugs.	[88]
CN102719362A	*Alternaria* sp.	Merlot grapes	Fungal strain capable of producing a large amount of resveratrol in the fermentation process.	[89]
CN102732427A	*Fusarium proliferatum*	*Oxytropis glabra*	Separation method for swainsonine-producing endophytic fungus.	[90]
CN102732428A	*Fusarium oxysporum*	*Cajanus cajan*	Endophytic fungal strain with a high yield of cajaninstilbene acid (59).	[91]
CN102787077A	*Acremonium* sp.	*Sophora alopecuroides*	Synthesis of matrine (60).	[92]
CN102807956A	*Ceriporia lacerata*	*Cleistocalyx operculatus*	Preparation of 2′,4′-dihydroxy-6′-methoxyl-3′,5′-dimethylchalcone (61).	[93]
WO2012020364A1	Fungal strain MTCC 5544	*Pongamia pinnata*	Dipeptide derivative (62) for the treatment of cancer.	[94]
CN103073527A	*Phomopsis* sp.	*Illigera rhodantha*	Preparation of libertellenone G (63) as a novel medicine for treating Alzheimer’s disease.	[95]
CN103074236A	*Trichoderma atroviride*	*Camptotheca acuminata*	Production and application of camptothecin.	[96]
CN103083290A	*Trichoderma* sp.	Not disclosed	*Trichoderma* acid (64) is involved in the preparation of antifungal agents.	[97]
CN103087923A	*Chaetomium globosum*	*Ginkgo biloba*	The endophytic fungus and metabolite flavipin (65) acts as an antioxidant.	[98]
CN103103134A	*Colletotrichum* sp.	*Huperzia serrata*	Production of huperzine A.	[99]
CN103194502A	*Nodulisporium sylviforme*	*Taxus* sp.	Separation and purification of taxol by biological fermentation as well as precursors such as baccatin III (66) and cephalomannine (67).	[100]
CN103288807A	Not disclosed	*Trypterigium wilfordii*	Separation of alkaloids (68–70) with pharmaceutical application.	[101]
CN103360351A	*Xylaria* sp.	*Azadirachta indica*	Obtaining three isopimarane diterpenoid compounds (71–73) with antifungal activity and potential applications in new agricultural or medical antifungal medicaments.	[102]
CN103436451A	*Colletotrichum* sp.	*Cyclocarya paliurus*	Production of haematochrome, including its production via a fermentation method.	[103]
IN2011DE03381A	*Diaporthe sp.*	*Pandanus amaryllifolius*	Antitubercular diaportheone B analogs (74–75) and their synthesis.	[104]
US20130137131A1	*Nodulisporium* sp., *Daldinia* sp., *Hypoxylon* sp.	*Persea indica*	System and method for producing volatile organic compounds	[105]
US20130177596A1	*Colletotrichum* sp.	*Pteromischum* sp.	Production of antifungal and immunosuppressive compounds	[106]
US20130224315A1	*Muscodor strobelli*	Not disclosed	Production of volatile organic compounds and methods of use	[107]
US20130252289A1	Several fungi such as *Nodulisporium* sp.*, Hypoxylon* sp.*, Annulohypoxylon* sp.*, Daldinia* sp.*, Xylaria* sp.	*Thelypteris angustifolia, Persea indica, Citrus aurantifolia, Myroxylon balsamum, Taxodium distichum*	Production of volatile organic compounds from microorganisms.	[108]
US20130302480A1	*Muscodor crispans*	*Ananas ananassoides*	Production of compounds with wide range of applications in agriculture, industrial, building, pharmaceutical and/or personal care products.	[109]
WO2013164834A1	*Fusarium solani*	*Taxus celebica*	Cost-effective process for commercial production of paclitaxel.	[110]
CN103570744A	*Scopulariopsis* sp.	*Carijoa sp.*	Preparation method for the quinazoline alkaloid compound (76) and its application as a tumor cell growth inhibitor.	[111]
CN103627736A	Fungal strain L1 CGMCC No. 4558	*Polygonum cuspidatum*	Extraction of resveratrol from fermented liquor.	[112]
CN103642864A	*Shiraia bambusicola*	*Huperzia serrata*	Preparation of hypocrellin compounds.	[113]
CN103667070A	*Trichoderma* sp.	*Huperzia serrata*	Preparation and application of huperzine A.	[114]
CN103667072A	*Ceriporia lacerata*	*Huperzia serrata*	Preparation of 8α, 15α-epoxy-huperzine A (77).	[115]
CN103667073A	*Peyronellaea glomerata*	*Huperzia serrata*	Preparation of pyrrole type (78) liver-protecting medicines.	[116]
CN103820331A	*Ceriporia lacerata/Hypoxylon investiens*	*Phlegmariurus* sp.	Production of huperzine A.	[117]
CN103820332A	*Pycnoporus sanguineus*	*Huperzia serrata*	Production of huperzine A.	[118]
CN103911293A	*Botryosphaeria dothidea*	*Taxus chinensis*	Strain with a high paclitaxel yield and method for producing paclitaxel.	[119]
CN103966109A	*Aspergillus fumigatus*	*Schisandra chinensis* fruit	Endophytic fungus that is capable of producing protocatechuic aldehyde (79).	[120]
CN104031948A	*Daldinia eschscholzii*	*Gracilaria* sp.	Production of dalesconol A (80) and B (81) as immunosuppressive compounds.	[121]
CN104059044A	*Trichoderma* sp./*Penicillium* sp.	Mangrove	Preparation of a xanthone derivative (82) as a microbial pesticide and fungicide.	[122]
CN104073529A	Not disclosed	*Taxus x media* seed	Production of taxol.	[123]
CN104086522A	*Lasiodiplodia pseudotheobromae*	*Camptotheca acuminata*	Preparation of a spiro-dinaphthalene compound (83).	[124]
CN104109691A	Not disclosed	*Ginkgo biloba*	Preparation and dyeing of red pigment haematochrome.	[125]
US20140082771A1	*Nodulosporium* spp. *or Ascocoryne* spp.	*Lomatia fraseri* or *Nothofagus cunninghamii*	Isolation of antibiotic compound.	[126]
CN104293678A	*Cladosporium cladosporioides*	*Forsythia* sp.	Production of forsythoside A (84), forsythoside B (85), and forsythin (86) and their applications.	[127]
CN104357525A	*Acremonium* *dichromosporum*	*Glycyrrhiza* sp.	Production of glycyrrhetinic acid by using microbial fermentation.	[128]
CN104450528A	Not disclosed	*Gardenia jasminoides*	Method for isolation and screening of endophytic fungi and for large-scale preparation of high-purity genipin (87).	[129]
CN104450531A	*Fusarium tricinctum*	*Fritillaria cirrhosa*	Obtains peiminine (88) and peimisine (89) alkaloids.	[130]
CN104593443A	*Botryosphaeria rhodina*	*Aquilaria sinensis*	Preparation of agilawood chromone (90–94) components.	[131]
CN104726345A	Mixtures of fungi including *Gliocladium* sp.	*Taxus* spp.	High production of baccatin III.	[132]
CN104762348A	Not disclosed	*Gastrodia elata*/*Armillaria mellea*	Preparation of gastrodin (95).	[133]
CN104774774A	*Aspergillus fumigatus*	*Glycyrrhiza* sp.	Production of pseutorin A (96) as a food preservative.	[134]
CN104789613A	*Alternaria* sp.	*Spiraea salicifolia*	Extraction and separation of bacteriostatic component (97) from fermentation broth.	[135]
CN104805017A	*Fusarium solani*	*Pinellia sp.*	Generation and application of β-glucosidase.	[136]
CN104877910A	*Eupenicillium brefeldianum*	Not disclosed	Preparation of brefeldin A (98). The compound has antifungal and insecticide activity and is an ideal veterinary and agriculture candidate drug.	[137]
CN105039173A	*Mortierella sp.*	*Huperzia serrata*	Fungal strain with a high huperzine A content.	[138]
CN105039174A	*Fusarium* sp.	*Paeonia* sp.	Production of paeonol (99).	[139]
CN105039175A	*Talaromyces* sp.	*Paeonia* sp.	Production of paeonol.	[140]
CN105039176A	*Fusarium* sp.	*Paeonia* sp.	Production of paeonol.	[141]
CN105200091A	*Geomyces sp.*	*Nerium indicum*	Production and application of ethyl vincamine (100).	[142]
US20150073048A1	*Muscodor* sp.	*Ananas ananassoides*	Production of antimicrobial composition and methods of use	[143]
WO2015029069A1	*Trichoderma longibrachiatum*	*Boswellia serrata*	Production of brachiatin D (101).	[144]
CN105238697A	*Chaetomium* sp.	*Paeonia* sp.	Production of paeonol with endophytic fungus from peony.	[145]
CN105238700A	*Epicoccum nigrum*	Wild soybean	High-yielding oleanolic acid endophyte.	[146]
CN105274005A	*Aspergillus fumigatus*	*Taxus x media*	Taxol production.	[147]
CN105316238A	*Trichoderma* sp.	*Taxus chinensis*	Method for culturing and screening taxol-producing fungus.	[148]
CN105349431A	*Phoma glomerata*	*Salvia miltiorrhiza*	Generation and application of salvianolic acid C (102).	[149]
CN105400842A	*Fusarium mairei*	*Taxus x media*/*Valeriana jatamansi*	Increases the yield of paclitaxel in an endophytic fungus fermentation product.	[150]
CN105505798A	*Phoma glomerata*	*Salvia miltiorrhiza*	Generation of ergosterol (103).	[151]
CN105506021A	*Aspergillus* sp.	Not disclosed	Preparation of taxol-containing culture.	[152]
CN105670940A	*Mucor racemosus*	*Huperzia serrata*	Application of a fungal strain with highly efficient expression of huperzine A.	[153]
CN105838613A	*Chaetomium globosum*	*Cajanus cajan*	Application of a fungal strain with a high yield of flavipin.	[154]
CN105925646A	*Phomopsis liquidambari*	Mangrove	Preparation method for cytochalasin H (104).	[155]
CN106010980A	*Paraconiothyrium brasiliense*	*Acrida cinerea*	Strain capable of producing of perlolyrine (105) and a method for preparation.	[156]
CN106047715A	*Trichoderma* sp.	*Nothapodytes pittosporoides*	Extraction of camptothecin.	[157]
WO2016034751A1	*Guignardia mangiferae*	*Persea indica*	Biocidal products (106) that are used to control phytopathogens and pest organisms.	[158]
CN106432168A	*Penicillium citrinum*	*Bruguiera sexangula* var. *rhynchopetala*	Preparation of isocoumarins (107–113) as antibacterial drugs.	[159]
CN106434361A	*Ascomycota* sp.	Mangrove	Preparation of indanone derivatives (114–115).	[160]
CN106497803A	*Fusarium verticillioides*	*Huperzia serrata*	Fungal strain with huperzine A-producing function and its use in the biosynthesis of medicine for treating Alzheimer´s disease and vascular dementia.	[161]
CN106497804A	*Fusarium oxysporum*	*Huperzia serrata*	Production of huperzine A and its application in the treatment of dementia.	[162]
CN106588944A	*Neonectria* sp.	*Meconopsis grandis*	Preparation of compound (116) derived from Tibetan medicine endophytic fungi.	[163]
CN106636247A	Not disclosed	*Melia azedarach*	Fermentation extraction of azadirachtin (117).	[164]
CN106701594A	*Neocosmospora* sp.	*Meconopsis grandis*	Production of pyrrocidine A (118) and pyrrocidine B (119).	[165]
CN106946955A	*Pezicula* sp.	*Taxodium distichum*	Production of mycotrisaccharide compounds (120–124) that aid in the preparation of drugs for preventing and controlling plant fungal disease.	[166]
CN106967622A	*Aspergillus flavus*	*Torreya fargesii*	Paclitaxel production.	[167]
CN106967623A	*Aspergillus niger*	*Torreya* sp.	Production of the taxane compound baccatin III.	[168]
CN106978356A	*Nigrospora sphaerica*	*Artemisia argyi*	Preparation of large amounts of bostrycin (125).	[169]
CN107034145A	*Pestalotiopsis vismiae*	*Cordyceps sinensis*	In vitro production of nucleosides, preferably, adenosine, guanylyl, uridine, and inosine.	[170]
CN107058118A	*Aspergillus aculeatus*	*Taxus x media*	Efficient taxol-producing endophytic fungus.	[171]
CN107118972A	*Epicoccum nigrum*	*Solidago canadensis*	Endophytic fungus capable of generating pectin through liquid fermentation.	[172]
CN107129936A	*Penicillium* sp.	*Torreya fargesii*	Production of paclitaxel.	[173]
CN107254504A	*Fusarium* sp.*/Bacillus aryabhattai*	*Erigeron breviscapus*	Increasing the scutellarin (126) content with microbial agents.	[174]
CN107354182A	*Purpureocillium lilacinum*	Grey green soy bean	Preparation of (R)-4-benzyl-2-oxazolidinone (127) by fermentation.	[175]
WO2017049353A1	*Daldinia* sp.	*Pittosporum bicolor*	Production of volatile organic compounds as insecticidal and antifungal agents.	[176]
WO2017068223A1	*Stemphylium solani*	*Artemisia absinthium*	To obtain compounds (128–129) for use as biocides.	[177]
CN107686817A	*Ascomycota sp.*	Fetid marsh fleabane	Production of ascomylactam compounds (130–131).	[178]
CN107723245A	*Fusarium sp.*	*Liriope spicata* var. *prolifera*	Endophytic fungi and application in the steroids saponin diosgenin (132) and ruscogenin (133).	[179]
CN107723246A	*Penicillium oxalicum*	*Liriope spicata* var. *prolifera*	Endophytic fungi and application in the steroid saponin diosgenin and ruscogenin.	[180]
CN107723247A	*Cladosporium sp.*	*Liriope spicata* var. *prolifera*	Endophytic fungi and application in the steroid saponin diosgenin and ruscogenin.	[181]
CN107723248A	*Penicillium* sp.	*Liriope spicata* var. *prolifera*	Endophytic fungi and application in the steroid saponin diosgenin and ruscogenin.	[182]
CN107739716A	*Penicillium* sp.	*Liriope spicata* var. *prolifera*	Endophytic fungi and application in the steroid saponin diosgenin and ruscogenin.	[183]
CN107739717A	*Schizophyllum* sp.	*Liriope spicata* var. *prolifera*	Endophytic fungi and application in the steroid saponin diosgenin and ruscogenin.	[184]
CN107739718A	*Aspergillus* sp.	*Liriope spicata* var. *prolifera*	Application in the preparation of the steroid saponin diosgenin and ruscogenin.	[185]
CN107868757A	*Bjerkandera adusta*	Not disclosed	Preparation of 8α, 15α-epoxy-huperzine A, which has a curative neuroprotective effect.	[186]
CN107955793A	*Aspergillus niger*	*Liriope spicata* var. *prolifera*	Preparation of the steroid saponin.	[187]
CN108264473A	*Penicillium decumbens*	Not disclosed	Preparation and application of 1-aniline-2-pyrrolidone class compounds (134–135).	[188]
CN108277164A	*Diaporthe* sp.	*Excoecaria agallocha*	Indene derivative (136) that aids in the preparation of an anti-inflammatory drug	[189]
CN108383811A	*Aspergillus tubingensis*	*Decaisnea fargesii*	Production of furanone (137) derivative with good antibacterial activity.	[190]
CN108467398A	*Trichoderma asperellum*	Seaweed	Preparation of diketopiperazine compound (138), which has antibacterial application.	[191]
CN108503616A	*Aspergillus tubingensis*	*Decaisnea fargesii*	Extraction method and application of a bicoumarin derivative (139).	[192]
CN108640897A	*Daldinia eschscholtzii*	Mangrove	Preparation and application of polyketides (140–141).	[193]
CN108728367A	*Phoma* sp.	Coral gorgonian source	Preparation of antibacterial compounds (142–143).	[194]
CN108913731A	*Pestalotiopsis* sp.	*Rhizophora stylosa*	Preparation and application of pestalotiopyrone M (144) which has immunosuppressive activity.	[195]
CN109082445A	*Fusarium proliferatum*	*Ginkgo sp.*	Production and application of glycine (145), betaine (146), scopoletin (147), yagaine, rosmarinic acid (148), oxipurinol (149), resveratrol, naringenin (150), catechin (151), taxifolin (152), and xanthohumol (153), which have antibacterial properties.	[196]
CN109096056A	*Aspergillus flavus*	*Kandelia obovata*	Preparation of bisabolane sesquiterpene compounds (154-155) as anti-type II diabetes mellitus drugs.	[197]
IN201641023516A	*Phomopsis* sp.	*Gloriosa superba*	Method of producing colchicine (156) from an endophyte using epigenetic modifiers.	[198]
IN201721003140A	*Phoma* sp.	*Litsea glutinosa*	Isolation, fermentation, purification, and characterization of the antibacterial compound 2’-hydroxygenistein (157).	[199]
CN109111422A	*Penicillium* sp.	*Panax notoginseng*	Macrolide compounds (158–167) and their application in the prevention and treatment of plant-pathogenic bacteria.	[200]
CN109180635A	*Xylaria curta*	*Solanum tuberosum*	Preparation and application of compound E1011 (168).	[201]
CN109206337A	*Fusarium* sp.	*Santalum album*	Method for preparation of hexichol phenolic acid compounds (169–171) and their application in the preparation of antibacterial compounds.	[202]
CN109232481A	Not disclosed	*Taxus chinensis*	Preparation of high-purity taxol.	[203]
CN109234175A	*Fusarium oxysporum*	*Paris polyphylla*	Production of chonglou saponin (172–175).	[204]
CN109265397A	*Lophiostoma* sp.	*Eucalyptus exserta*	Fast separating process of fungal secondary metabolites (176–177).	[205]
CN109293494A	*Talaromyces* sp.	Mangrove	Method for preparation of 1, 4-naphtoquinone compounds (178–179) and their application in the preparation of anti-inflammatory drugs.	[206]
CN109439705A	*Aspergillus* sp.	Soft coral	Microbe preparation of subergorgic acid (180).	[207]
CN109456191A	*Cerrena sp.*	*Pogostemon cablin*	Preparation of cerrenin D (181) that is applied in the preparation of antitumor drugs.	[208]
CN109456899A	*Penicillium notatum*	*Gastrodia elata*	Fermentation and production of penicillic acid (182).	[209]
CN109486685A	*Penicillium* sp.	Mangrove	Preparation of anti-insect activity terpenes (183–184) as crystalline compounds.	[210]
CN109503414A	*Trichoderma asperellum*	Seaweed	Preparation of one kind of alkane sesquiterpene derivative (185).	[211]
CN109503428A	*Trichoderma asperellum*	Seaweed	Preparation of a cyclonerolane-type hydroxamic acid derivative (186).	[212]
CN109503535A	*Trichoderma asperellum*	Seaweed	Preparation of a bicyclic cyclonerolane type sesquiterpene derivative (187).	[213]
CN109503623A	*Trichoderma koningiopsis*	*Morinda officinalis*	Preparation and application of guanacaste class compounds (188–189) in the preparation of antibacterial compounds.	[214]
CN109553600A	*Penicillium* sp.	Mangrove	Preparation and application of isocoumarin class compounds (190–197).	[215]
CN109651125A	Fungal strain ZJY1288 GDMCC No. 60290	Mangrove	Preparation and application of anthraquinone metabolites (198–199)	[216]
CN109776561A	*Cytospora rhizophorae*	*Morinda officinalis*	Preparation of cytorhizin B (200) and C (201) that are applied in the preparation of antitumor drugs.	[217]
CN109810906A	*Bionectria pityrodes*	*Tamarix* sp.	Preparation of phenolic acid compound (202) through fermentation.	[218]
CN109956883A	*Trichoderma asperellum*	Seaweed	Preparation of an azo-cyclo alkane type sesquiterpene derivative (203) produced through an acetylation method.	[219]
CN109971652A	*Onygenales* sp.	*Incarvillea younghusbandii*	Preparation of gymnoascolide A (204) in preparing anti-inflammatory drugs.	[220]
CN109971651A	*Arthrinium arundinis*	Tobacco	Preparation of 5, 8-peroxyde of ergosterol.	[221]
CN109971655A	*Chaetomium* sp.	Radix astragali	Production of differanisole A (205).	[222]
CN109988181A	*Bipolaris* sp.	*Lycium barbarum*	Preparation of bipolahydroquinone C (206) that is used as an antineoplastic drug for treating human pulmonary squamous carcinoma and breast carcinoma.	[223]
CN110093383A	*Alternaria* sp.	*Polygonum senegalense*	Preparation of compound alterlactone (207) that is used as a disinfectant in agriculture.	[224]
CN110218200A	*Pseudopithomyces* sp.	*Sonneratia caseolaris*	Preparation of depsipeptide compound (208).	[225]
CN110229127A	fungal strain TGM112 CGMCC No. 16499	Mangrove	Preparation of butyrolactone compounds (209–211).	[226]
CN110257255A	*Daldinia eschscholtzii*	Mangrove	Preparation of chromone derivatives (212–216).	[227]
CN110257260A	*Boeremia exigua*	*Atractylodis macrocephalae*	Preparation of the *Atractylodes* lactones I (217) and II (218).	[228]
CN110272828A	*Colletotrichum boninense*	*Huperzia serrata*	New microbe resource for the production of huperzine A industrial fermentation.	[229]
CN110283728A	*Daldinia eschscholtzii*	Mangrove	Preparation of tetralone derivatives (219–223).	[230]
CN110295116A	*Aspergillus* sp.	*Tamarix sp.*	Production of a variety of fatty acids and their application.	[231]
CN110302215A	*Penicillium* sp.	*Taxus x media*	Fungal crude extract, it‘s applications, e.g., as being a source of paclitaxel analog.	[232]
CN110438015A	*Aspergillus tamarii*	Citron orange fruit	Fungal strain its fermentation to produce hesperidinase.	[233]
CN110484588A	*Acremonium pilosum*	*Mahonia sp*.	Preparation of fusidic acid (224).	[234]
CN110511876A	*Ilyonectria cyclaminicola*	Korean *Epimedium* herb	The culture method of this fungal strain and its metabolites epimedins A–C (225–227).	[235]
CN110563740A	*Aspergillus fumigatus/Fusarium oxysporum*	*Edgeworthia chrysantha*/*Stachys japonica*	Methods for preparation and application of alpha-pyrone (228).	[236]
IN201721002537A	*Aspergillus japonicus*	*Achryranthes aspera*	Production of the novel antibacterial compound fraxidin (229).	[237]

^1^ Some patents just provided a common name for the host organism.

**Table 2:** Endophytic fungi applied for biotransformation.

Patent No.	Endophyte	Host ^1^	Patent Details	Ref.
CN102080048A	*Absidia glauca*	gingsen	Conversion of ginsenoside Rb1 (230) to prepare ginsenoside Rd (231).	[245]
CN102080049A	*Zygorhynchus moelleri*	*Panax gingsen*	Preparation of ginsenoside Rd from ginsenoside Rb1.	[246]
CN102154123A	*Fusarium* sp.	*Dioscorea nipponica*	Biotransformation conversion conditions of diosgenin saponins.	[247]
CN102199548A	*Penicillium oxalicum*	*Polygonun cuspidatum*	Microbial transformation of resveratrol from polydatin (232).	[248]
CN102212486A	*Penicillium oxalicum*	*Polygonun cuspidatum*	Conversion of polydatin into resveratrol.	[249]
CN102392050A	*Penicillium* sp.	Not disclosed	Biotransformation of raisin extract. Preparation and application in flavoring.	[250]
CN102757443A	Several endophytes featuring *Penicillium purpurogenum*	*Dysosma* sp. *or Sabina vulgaris*	Separation and purification method for bioconversion of podophyllotoxin into sulfur-substituted derivatives.	[251]
CN103695478A	fungal strain L1 CGMCC No. 4558	Not disclosed	Conversion of polydatin to resveratrol.	[252]
CN103981104A	*Microsphaeropsis arundinis*	wild rice	Biotransformation of glycyrrhizinic acid (233) into liquiritin (234).	[253]
CN103992953A	*Aspergillus flavus*	wild rice	Transform glycyrrhizic acid into glycyrrhetinic acid monoglucuronide (235).	[254]
CN106591142A	*Xylariales* sp.	Not disclosed	Conversion of *Panax notoginseng* saponin to prepare vina-ginsenoside R13 (236), notoginsenoside J (237) and American saponin ginseng L16 (238).	[255]
CN106701604A	*Chaetomium globosum*	wild rice	Conversion of glycyrrhizic acid into glycyrrhetinic acid monoglucuronide.	[256]
CN106893677A	*Fusarium* sp.	Herba Andrographitis	Transformation of andrographolide diterpenoids (239–242).	[257]
CN107034253A	*Fusarium oxysporum*	*Gentiana* sp.	Conversion of gentiopicroside (243) into two separate compounds with hepatoprotective activity.	[258]
CN107312720A	*Fusarium proliferatum*	*Cajanus cajan*	Conversion of ginsenoside Rb1 into ginsenoside Rd and its application.	[259]
CN108707553A	*Plectosphaerella cucumerina*	*Huperzia serrata*	Efficient conversion of androstenedione to testolactone and androstane diene diketone.	[260]
CN109536561A	*Fusarium oxysporum*	gingsen	Conversion of ginsenoside Rb1 into the rare ginsenoside CK (244).	[261]
CN110527632A	*Phomopsis* sp.	Not disclosed	Bioconversion of betulinic acid (245).	[262]
CN110423697A	*Lasiodiplodia pseudotheobromae*	*Illicium verum*	*trans*-*trans*-Anethole (246) conversion to generate different vanillic acids (247).	[263]
US20190264295A1	*Ovatospora brasiliensis*	*Curcuma* sp.	Microbial bioconversion of curcuminoids to calebin A (248).	[264]
WO2019070219A2	*Alternaria eureka/Neosartorya hiratsukae/Camarosporium laburnicola*	*Astragalus condensatus*, *A. angustifolius*	Production of a telomerase activator, biotransformation with endophytic fungi to obtain new/novel molecules from the saponins from natural sources and method for discovery molecules that increase telomerase enzyme activation.	[265]

^1^ Some patents just provided a common name for the host organism.

## Supplementary Materials

The following are available online at https://www.mdpi.com/2309-608X/6/2/58/s1, Figure S1: Structures of the secondary metabolites listed in Table 1 and Table 2.
