# Supplementary material for: Patents on Endophytic Fungi Related to Secondary Metabolites and Biotransformation Applications
Source: J Fungi (Basel). 2020 May 1;6(2):58. doi: 10.3390/jof6020058 (PMC7344749; doi:10.3390/jof6020058)
Supplement: Supplementary file 1 [file jof-06-00058-s001.pdf]

Supplementary data

Patents on endophytic fungi related to secondary metabolites and biotransformation applications

Daniel Torres-Mendoza[1, 2], Humbeto E. Ortega[1, 3], Luis Cubilla-Rios[1*]

1 Laboratory of Tropical Bioorganic Chemistry, Faculty of Natural, Exact Sciences and Technology, University of Panama, Panama.

2 Vicerrectoría de Investigación y Postgrado, University of Panama, Panama.

3 Department of Organic Chemistry, Faculty of Natural, Exact Sciences and Technology, University of Panama, Panama.

Figure S1. Structures of the secondary metabolites listed in Table 1 and 2

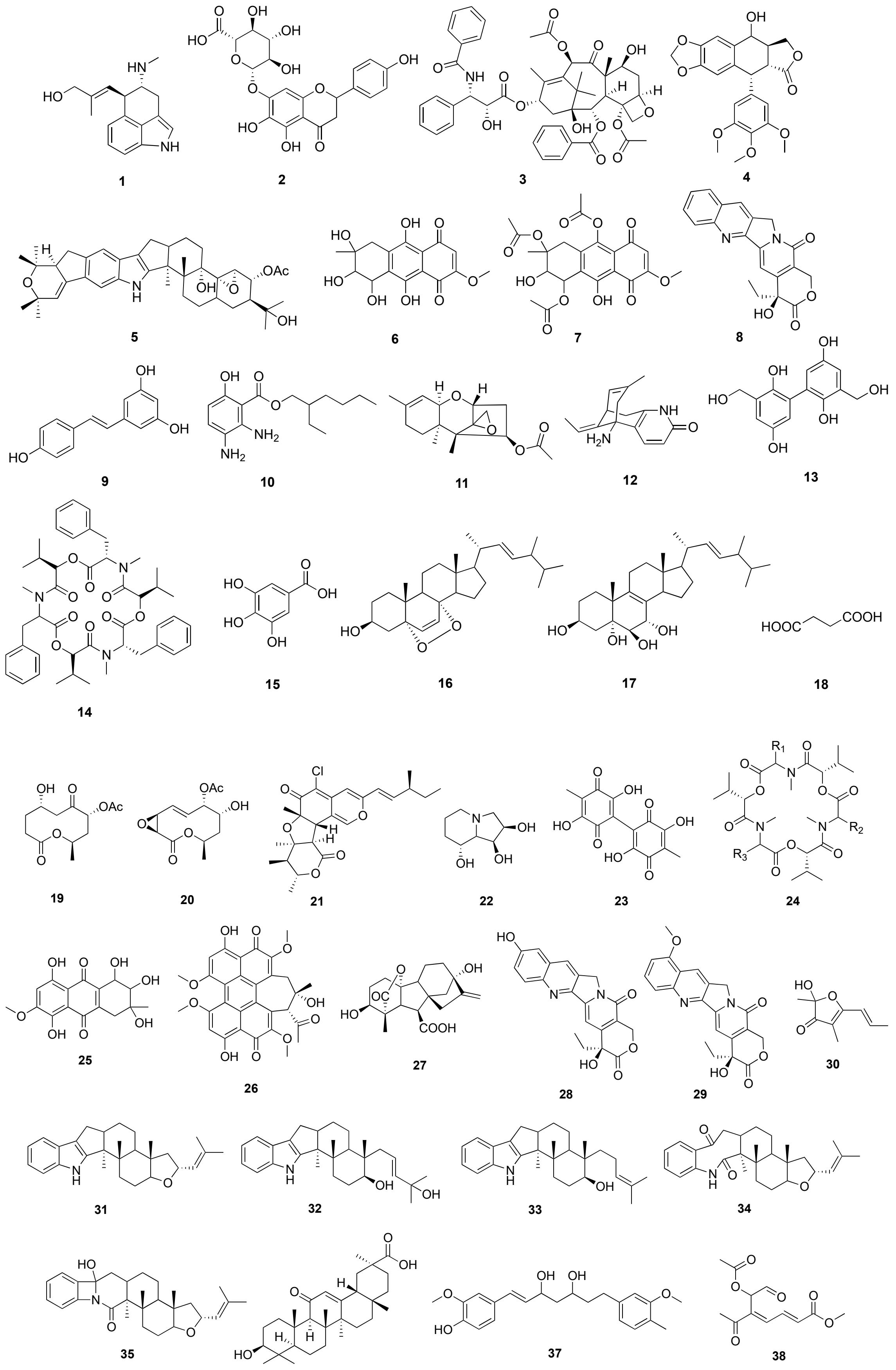

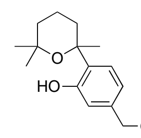

39

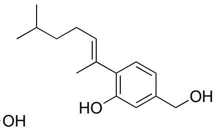

40

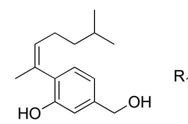

41

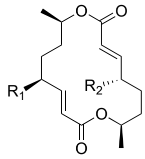

42 $R_1 = R_2 = OH$
43 $R_1 = OH, R_2 = {=}O$
44 $R_1 = R_2 = {=}O$

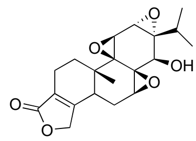

45

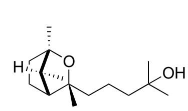

46

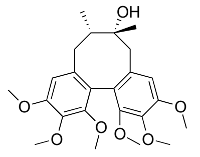

47

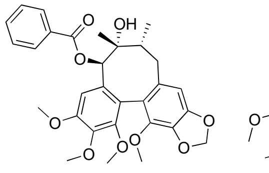

48

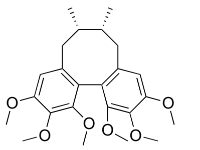

49

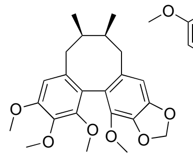

50

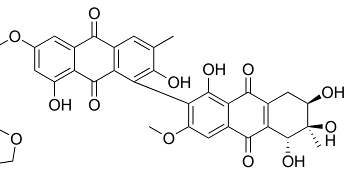

51

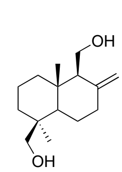

52

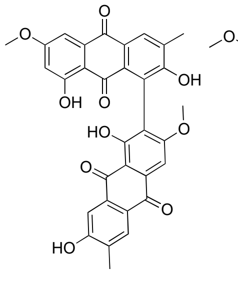

53

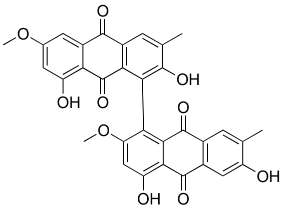

54

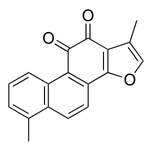

55

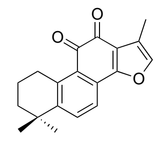

56

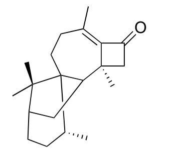

57

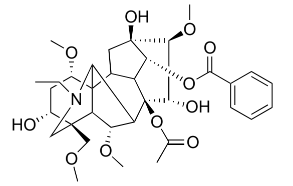

58

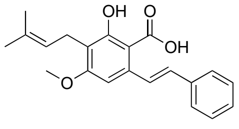

59

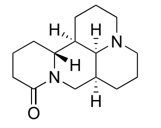

60

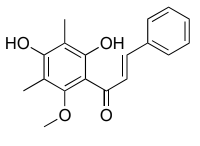

61

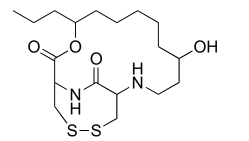

62

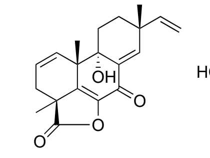

63

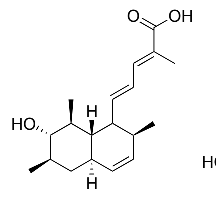

64

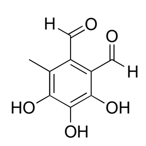

65

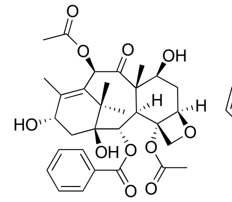

66

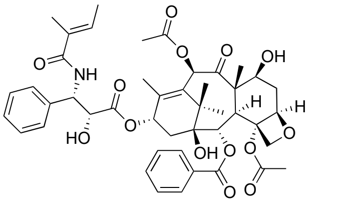

67

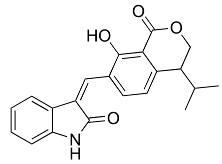

68

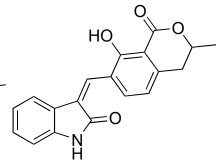

69

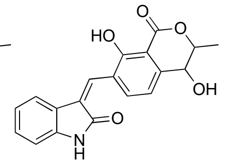

70

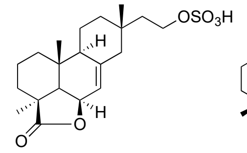

71

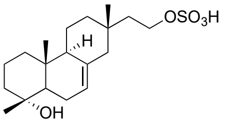

72

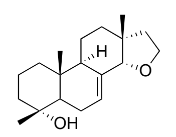

73

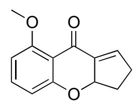

74

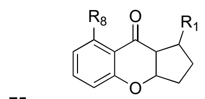

75
R_1 = H or (C_1-C_6)alkyl or OH or C=O
R_8 = H or OH or (C_1-C_6)alkoxy

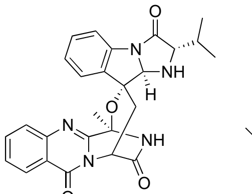

76

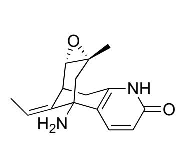

77

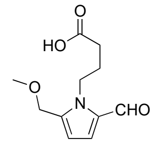

78

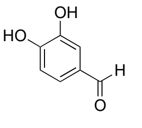

79

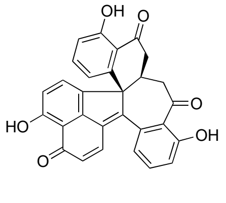

80

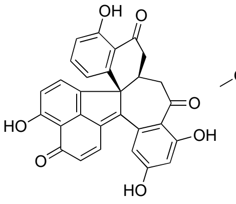

81

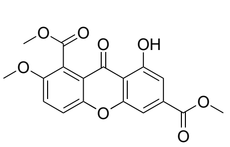

82

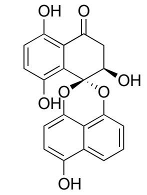

83

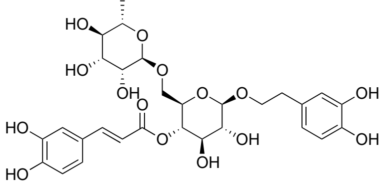

84

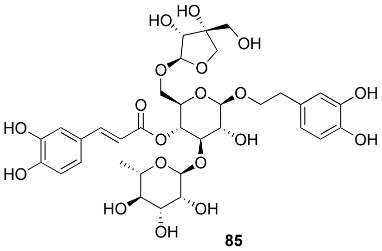

85

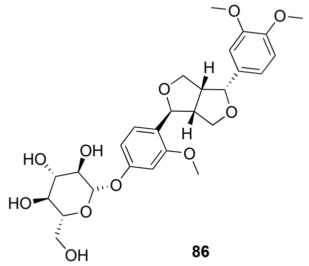

86

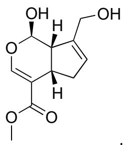

87

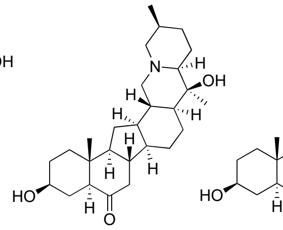

88

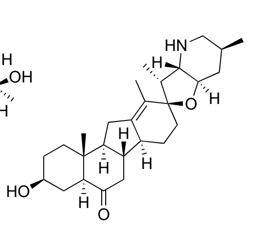

89

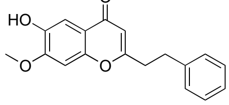

90

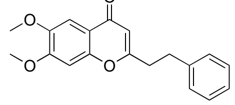

91

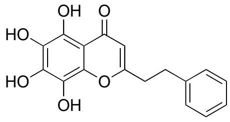

92

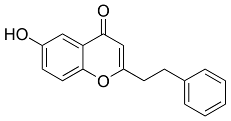

93

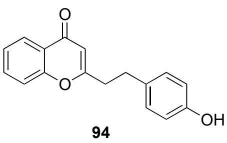

94

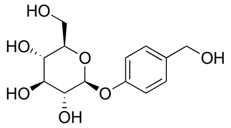

95

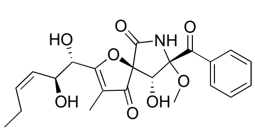

96

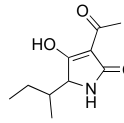

97

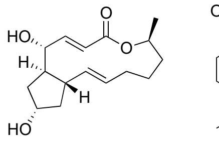

98

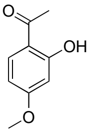

99

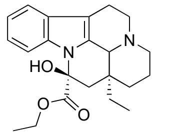

100

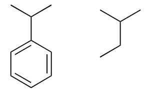

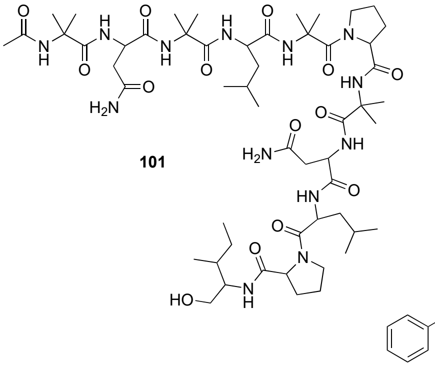

101

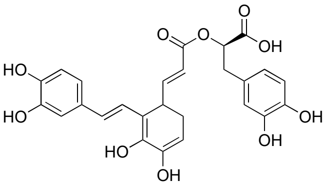

102

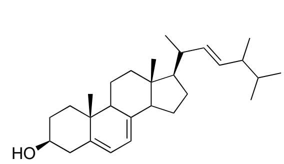

103

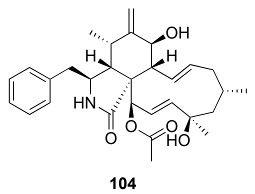

104

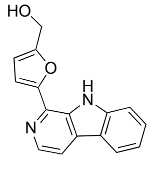

105

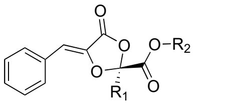

106

I **R_1 = *sec*-butyl; R_2 = H**

II **R_1 = *sec*-butyl or methyl-phenyl;**
R_2 = CH_3 or H or ethyl-phenyl

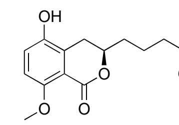
107
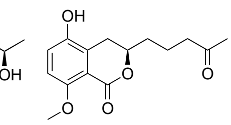
108
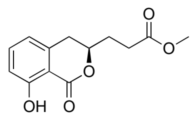
109
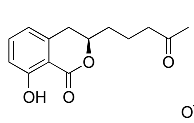
110
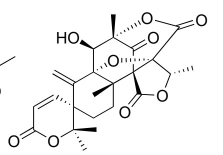
111

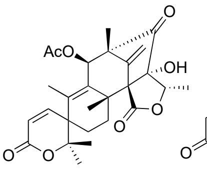
112
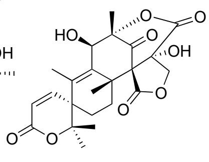
113
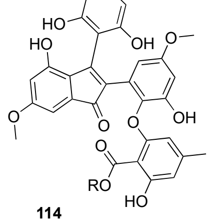
114
R = H or Me
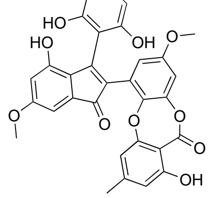
115
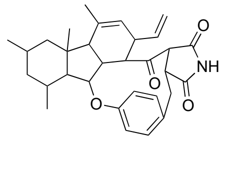
116

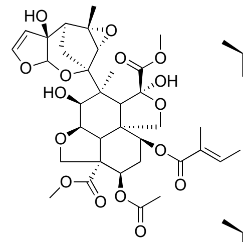
117
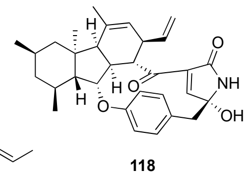
118
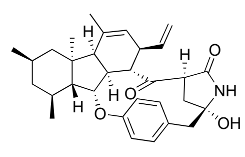
119
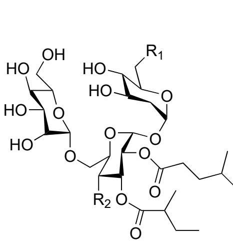
120 R_1 = H; R_2 =
121 R_1 = H; R_2 =
122 R_1 = H; R_2 =
123 R_1 = H; R_2 =
124 R_1 = ; R_2 =
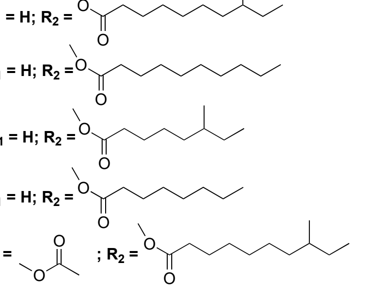

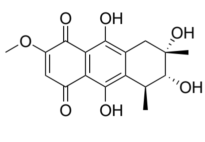
125
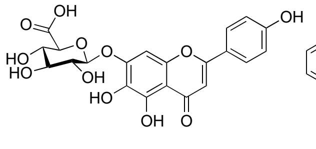
126
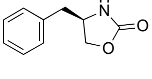
127
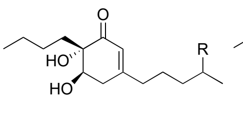
128
R = H or OH
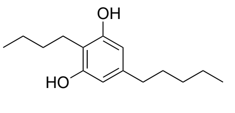
129

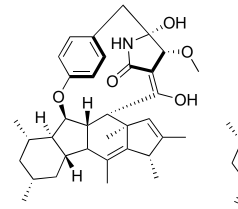
130
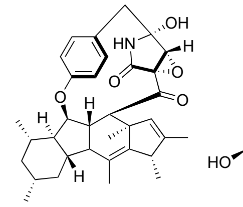
131
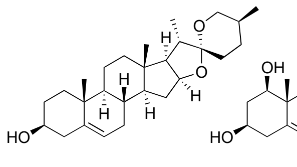
132
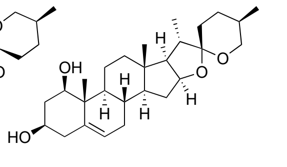
133
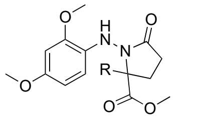
134 R = OCH_3
135 R = OH

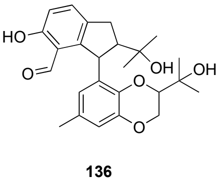
136
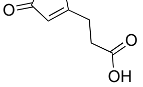
137
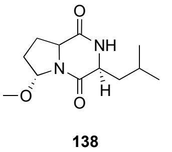
138
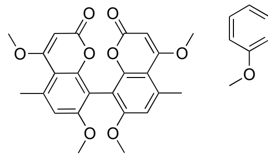
139
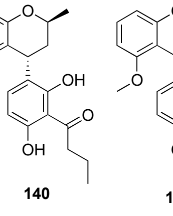
140
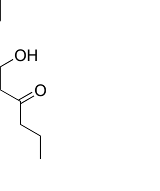
141

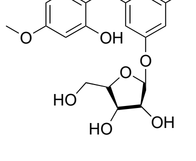
142
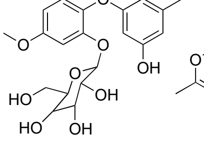
143
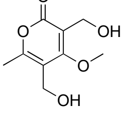
144
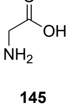
145
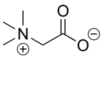
146
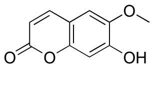
147
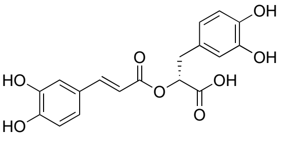
148

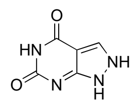
149

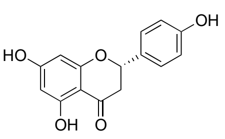
150

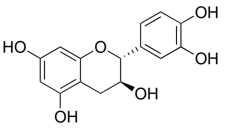
151

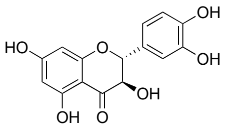
152

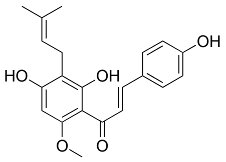
153

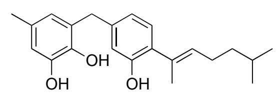
154

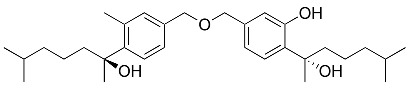
155

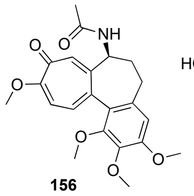
156

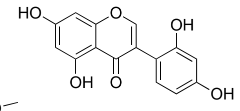
157

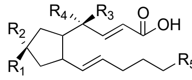

158 $R_1 = R_2$ = =O, R_3 = H, R_4 = OH, R_5 = OAc
159 R_1 = OAc, $R_2 = R_3$ = H, R_4 = OH, $R_5 = COCH_3$
160 R_1 = OAc, $R_2 = R_4$ = H, R_3 = OH, $R_5 = COCH_3$
161 $R_1 = R_3$ = H, $R_2 = R_4$ = OH, $R_5 = CH_3CHOAc$
162 $R_1 = R_4$ = OH, $R_2 = R_3$ = H, $R_5 = CH_3CHOAc$

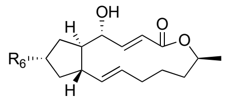

163 R_6 = OH
167 R_6 = OAc

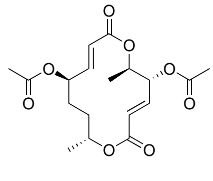
168

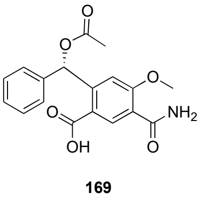
169

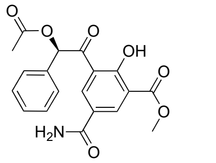
170

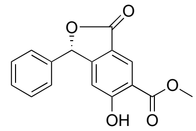
171

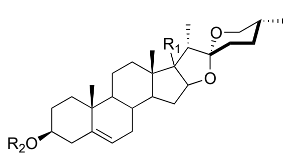

172 R_1 = H, R_2 = Rha(1→2)-Ara(1→4)-Glc
173 R_1 = H, R_2 = Rha(1→4)-Rha(1→4)[Rha(1→4)]-Glc
174 R_1 = OH, R_2= Rha (1→2)-Glc
175 R_1 = OH, R_2 = Rha(1→4)-Rha(1→4)[Rha(1→2)]-Glc

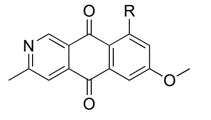

176 R = OH
177 R = OCH_3

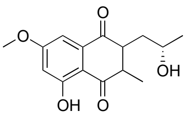
178

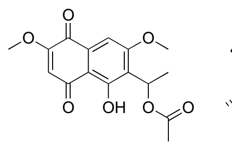
179

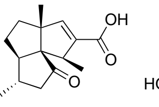
180

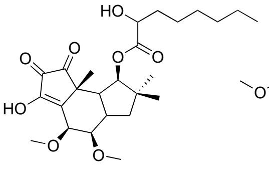
181

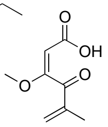
182

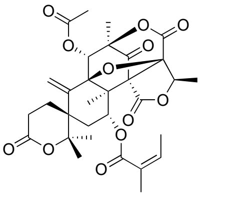
183

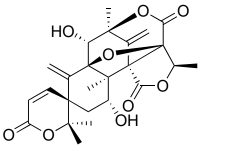
184

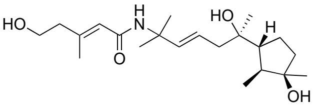
185

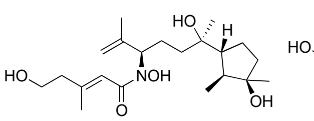
186

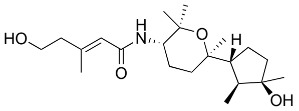
187

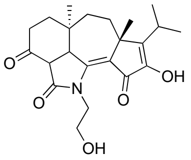
188

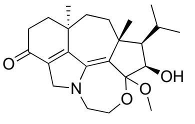
189

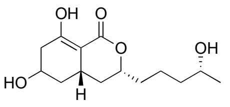
190

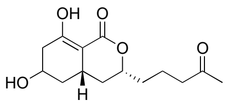
191

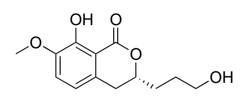
192

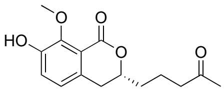
193

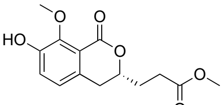
194

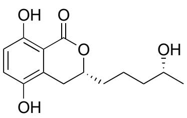
195

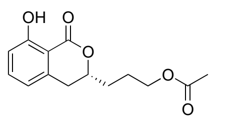
196

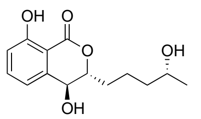
197

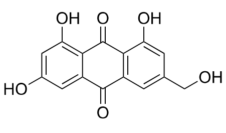
198

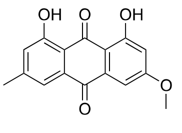
199

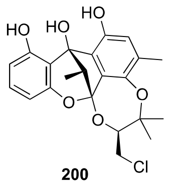
200

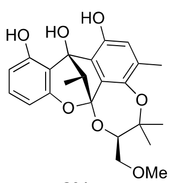
201

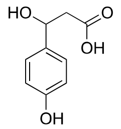
202

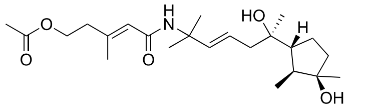
203

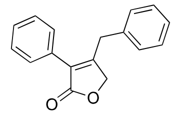
204

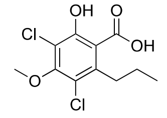
205

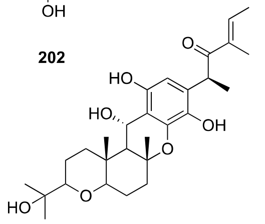
206

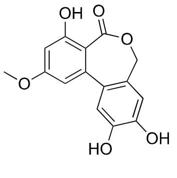
207

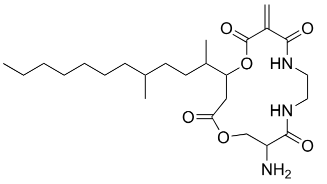
208

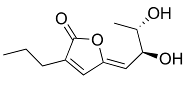
209

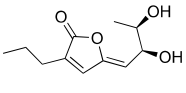
210

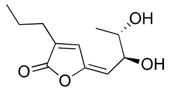
211

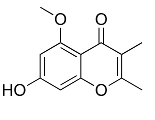
212

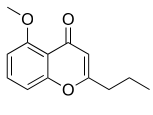
213

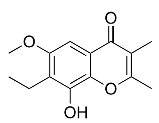
214

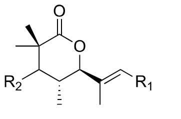
215 **R_1 = CH_2OH, R_2 = a-OH**
216 **R_1 = CH_2OAc, R_2 = a-OH**

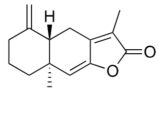
217

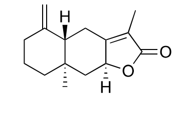
218

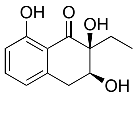
219

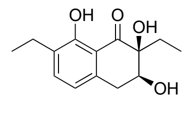
220

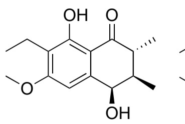
221

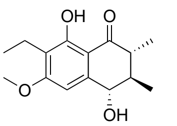
222

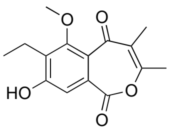
223

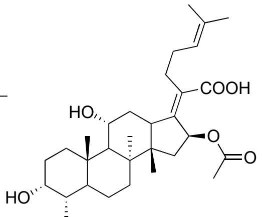
224

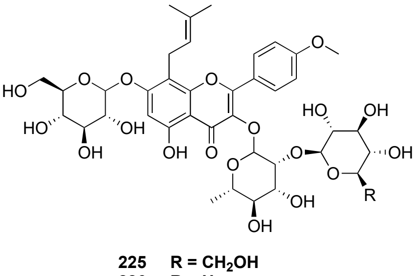
225 **R = CH_2OH**
226 **R = H**
227 **R = CH_3**

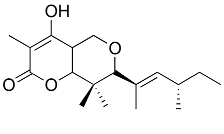
228

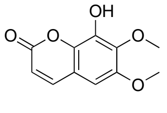
229

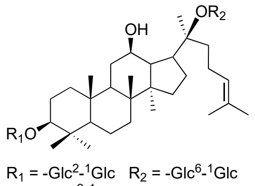

230 R_1 = -Glc[2]-[1]Glc R_2 = -Glc[6]-[1]Glc
231 R_1 = -Glc[2]-[1]Glc R_2 = -Glc

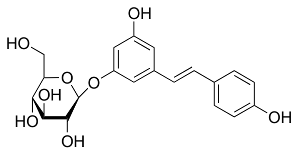

232

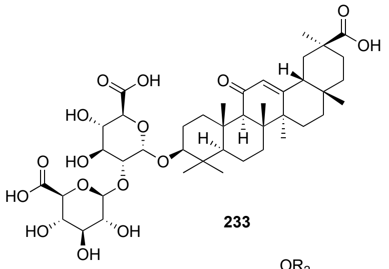

233

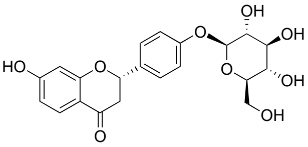

234235

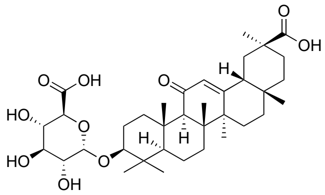

235

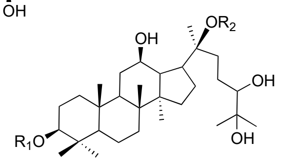

236 R_1 = -Glc[2]-[1]Glc R_2 = -Glc
238 R_1 = -Glc[2]-[1]Glc R_2 = -Glc[2]-[1]Glc

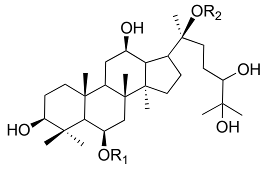

237 R_1 = R_2 = -Glc

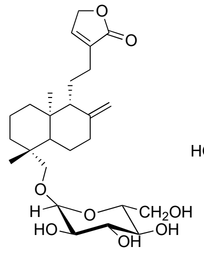

239

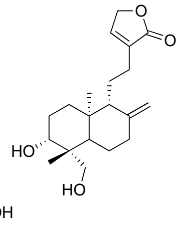

240

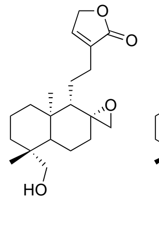

241

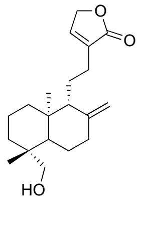

242

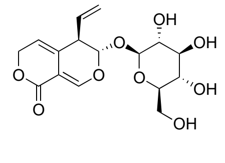

243

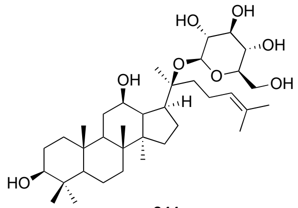

244

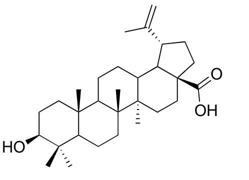

245

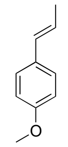

246

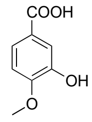

247

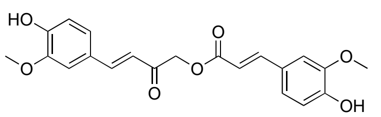

248
